# “Is It Overtraining or Just Work Ethic?”: Coaches’ Perceptions of Overtraining in High-Performance Strength Sports

**DOI:** 10.3390/sports9060085

**Published:** 2021-06-07

**Authors:** Lee Bell, Alan Ruddock, Tom Maden-Wilkinson, Dave Hembrough, David Rogerson

**Affiliations:** Department of Sport and Physical Activity, Sheffield Hallam University, Sheffield S10 2BP, UK; a.ruddock@shu.ac.uk (A.R.); t.maden-wilkinson@shu.ac.uk (T.M.-W.); d.hembrough@shu.ac.uk (D.H.); d.rogerson@shu.ac.uk (D.R.)

**Keywords:** overtraining syndrome, overreaching, functional overreaching, strength training, resistance training

## Abstract

Optimal physical performance is achieved through the careful manipulation of training and recovery. Short-term increases in training demand can induce functional overreaching (FOR) that can lead to improved physical capabilities, whereas nonfunctional overreaching (NFOR) or the overtraining syndrome (OTS) occur when high training-demand is applied for extensive periods with limited recovery. To date, little is known about the OTS in strength sports, particularly from the perspective of the strength sport coach. Fourteen high-performance strength sport coaches from a range of strength sports (weightlifting; *n* = 5, powerlifting; *n* = 4, sprinting; *n* = 2, throws; *n* = 2, jumps; *n* = 1) participated in semistructured interviews (mean duration 57; SD = 10 min) to discuss their experiences of the OTS. Reflexive thematic analysis resulted in the identification of four higher order themes: *definitions*, *symptoms, recovery* and *experiences and observations*. Additional subthemes were created to facilitate organisation and presentation of data, and to aid both cohesiveness of reporting and publicising of results. Participants provided varied and sometimes dichotomous perceptions of the OTS and proposed a multifactorial profile of diagnostic symptoms. Prevalence of OTS within strength sports was considered low, with the majority of participants not observing or experiencing long-term reductions in performance with their athletes.

## 1. Introduction

In sports such as weightlifting and powerlifting, the goal is to successfully lift the largest mass within a weight class [1]. Sports that involve maximal effort throwing, jumping, and sprinting are determined by mass-specific force generation and impulsiveness [2]. Optimal performance in these sports is achieved using planned periods of strength training and recovery with the aim of inducing physiological adaptations that underpin performance [3]. This is typically achieved by strategically organising training load to achieve peak performance at key periods within the competition schedule [4].

Short-term, intentional periods of increased training (multiple daily training sessions, increases in volume and intensity) have been used in weightlifting [5,6], powerlifting [7,8] and track and field sports [9] to induce a performance “supercompensation” or “rebound” effect, typically observed within approximately <10 (2–5) weeks after the resumption of normal or reduced training. Importantly, this occurs after an initial relative reduction in performance [5]. This method has been referred to as functional overreaching (FOR) [10]. Whilst FOR might occur if the balance between training and recovery is achieved, there is a risk that prolonged training without enough recovery can impair adaptation and long-term performance and result in either nonfunctional overreaching (NFOR), which can take several weeks to recover from, or the overtraining syndrome (OTS), where recovery can take several months or longer [10]. The terms “staleness”, “fatigue syndrome” and “unexplained underperformance syndrome” have all been used interchangeably with OTS [11]. NFOR has been referred to as “overreaching” (OR) in the literature, whilst the terms “overtraining” (OT) and “underrecovery” have been used to describe the imbalance between intensified training and insufficient recovery which can lead to OTS [10,11].

Evidence for the identification, diagnosis, and prevalence of OTS in strength sports or resistance training is limited [6,11,12,13,14]. Whilst more than 70% of strength sport competitors have reported unexplained decreases in performance, approximately 43% of those have indicated a range of symptoms lasting one week to one month, with 13.1% lasting one to three months and only 4.7% > 4 months [15], suggesting higher occurrence of acute fatigue or OR, and lower incidence of OTS.

A lack of individualised testing protocols and gold standard markers combined with multidimensional and individual symptoms make the identification of OTS difficult [10,15]. As such, there are a lack of reliable, prognostic tools for sport coaches to accurately judge when periods of increased training will lead to performance supercompensation or result in NFOR/OTS. Currently, OTS can only be assessed retrospectively using the diagnostic flowchart presented by the joint consensus statement of the European College of Sport Science (ECSS) and the American College of Sports Medicine (ACSM) [10]. However, the nature of such a tool suggests it can only be utilised by those already considered to be at risk of OTS. Further, there is limited agreement on “textbook” symptoms in the overtrained athlete, with athletes either presenting a myriad of individualised symptoms, exhibiting normal symptoms of acute fatigue, or displaying an asymptomatic profile [10]. Current guidance on management of symptoms and diagnostic assessment of OTS has been developed largely from studies from the endurance sport domain [10,16] and fewer from strength sports or resistance training populations [12,13,14]. However, the profile of endurance and strength athletes exhibiting symptoms of OTS typically elicit different responses [13], making the identification and management of NFOR/OTS difficult for the strength coach.

It is important to improve communication between laboratory research and applied experience in order to develop robust and practical coaching tools, particularly in under-investigated area such as OR/OTS, and where the existing literature suggests inconsistency between expert statements, experimental study findings and the application of such evidence to the training environment [10,12,13,14,15]. To our knowledge, there has been no previous qualitative research undertaken to capture high-performance strength sport coaches’ perceptions of long-term performance decline and their experiences of OTS. Such research could elicit important information about the understanding of OTS, its impact on strength athletes, and approaches to managing the delicate balance between training and recovery. The aim of this study therefore was to explore perceptions of OT and OTS in high-performance strength coaching, and to provide a new way of understanding and conceptualising these concepts.

## 2. Materials and Methods

### 2.1. Study Design

To answer the aims of this study, semistructured interviews were analysed using reflexive thematic analysis based on guidelines provided by Braun and Clarke [17,18]. Thematic analysis is a method for identifying, organising, analysing, and reporting qualitative data sets into compressed meaningful patterns [19], and provides a suitable design when examining lived experiences [20]. Thematic analysis is becoming an increasingly common tool for qualitative research within sport and exercise science, however, its use within this domain has previously been criticised for misapplication of its methodological principles [18]. Therefore, following detailed theoretical and conceptual frameworks such as the one provided by Braun and Clarke [17] is fundamental to better thematic analysis practice [17,18].

### 2.2. Participants

Following institutional ethical approval (ER16222001), 14 high-performance strength sport coaches were recruited via opportunity sampling. All benefits and risks were explained prior to data collection, informed consent was obtained, and the study was conducted according to the principles of the Declaration of Helsinki. Inclusion criteria stated that participants had a minimum of 3 years’ experience of coaching in at least one strength sport at a national level or above. For the purpose of this research, *strength sports* were defined as either weightlifting, powerlifting, sprinting, jumps (e.g., long jump, triple jump) or throwing sports (e.g., hammer, discus, javelin). Sample size was determined by the principal of saturation, with participants recruited until new data failed to evolve further insight or provide novel information [21]. Based on previous research guidance [21], a nonprobabilistic sample size of ≥ 6 participants was expected to achieve saturation due to the participant pool being recruited from a homogenous group.

Participants comprised 12 males and 2 females (the descriptive profile of each participant is located in Table 1). Duration of experience ranged from 4 to 57 years (mean 14.4; SD = 13.4 years) and participants included a cross-section of strength sports: weightlifting (5), powerlifting (4), and track and field (5). Track and field coaches consisted of sprinting (2), jumps (1), and throws (2). Ten participants were based in the United Kingdom, 2 in Republic of Ireland, 1 in the United States and 1 in New Zealand. Education level ranged from no academic degree to Doctorate of Philosophy. Coaches possessed a range of relevant governing body certifications for their respective sport, with some holding additional strength and conditioning accreditation.

To maximise the quality and range of potential candidates, initial contact was made with relevant national governing bodies via email. An information and recruitment poster was provided at initial contact, detailing the aims of the research study, as well as inclusion and exclusion criteria. After, an information and recruitment poster was shared organically across several social media platforms, and additional sharing of the poster was encouraged across social media to widen the reach of the study. The research team also shared the poster via personal and institutional social media channels. A supplementary participant information sheet was presented to potential participants after initial contact had been made to provide further details about the interview process. Each interested participant was screened by the principal investigator (L.B.) prior to the interview to ensure inclusion criteria were met.

### 2.3. Procedures

**Figure 1:**
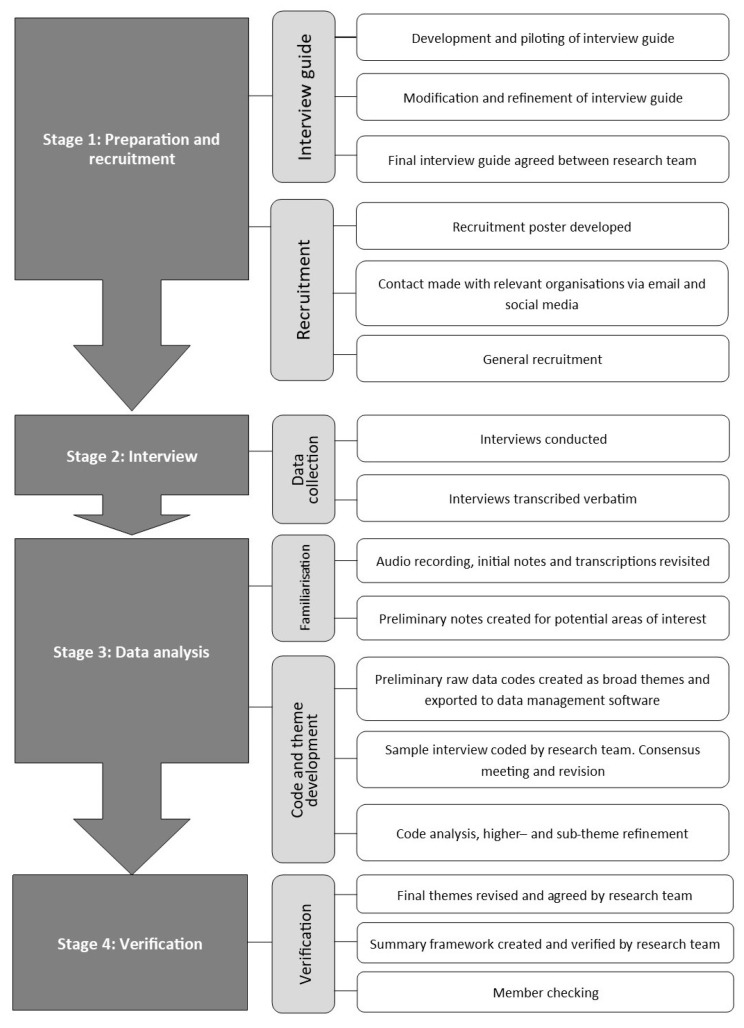
Flow diagram of data collection procedure.

Before data collection, an interview guide was created by the principal investigator in consultation with the research team and refined through a process of pilot interviewing. Pilot interviews were conducted on two strength coaches. The interview guide was further developed after piloting to reflect the aims and objectives of this study. A semistructured interview approach permitted the collection of rich data whilst remaining focused on the study objectives [22], and provided a qualitative method previously used within strength and conditioning research to ascertain coaches’ experiences [23]. All interviews were conducted by the principal investigator. A flow diagram of the data collection procedure is presented in Figure 1.

Participants were invited to either a face-to-face or online interview based on geographical location and availability. Face-to-face interviews were conducted in a neutral, quiet, and mutually agreed environment, and data were collected using a digital voice recorder (Zoom, Hn1 digital recorder 2.0, UK). For online interviews, data were collected using European Union General Data Protection Regulation-compliant software (Skype Ltd, version 15, Luxembourg) and exported to a password-protected external hard drive (Seagate Technology PLC, Fremont, CA, USA). All data were anonymised, and only participant identification numbers were used during publication of results, assigned chronologically based on order of interview. The principal investigator sought to conduct interviews that built trust through relaxed dialogue and rapport building, and actively explored participant responses in a systematic and comprehensive way. To achieve this, the interview agenda was approached in a fluid and flexible manner; an important aspect of realist interview good practice [24].

Throughout the interview, participants were encouraged to draw upon their own experiences, offer detailed and practical responses where possible, and to elaborate where appropriate to provide rich, experiential responses. Introductory questions were used to provide descriptive and contextual background information, and to act as an “ice breaker” between participant and researcher (for example, *“tell me a bit about yourself and what sport you’re involved in”*). Central questioning related to the research question (for example, *“how would you define the overtraining syndrome?”*). Closing questions provided participants with an opportunity to reflect or add anything they thought relevant to the interview that had not already been covered (*“before we finish, is there anything you’d like to elaborate on or add to the discussion?”*). The principal investigator created field notes throughout the interview to act as prompts for additional questioning and to explore lines of enquiry not contained in the interview guide.

### 2.4. Data Analysis

The initial stage of analysis involved immersion in the overall dataset by repeated listening to recordings and reading of transcripts and highlighting potential “points of interest” using Google Document highlight colour tools. During this initial phase, potential data codes were identified by extracting lines and/or paragraphs deemed relevant to the research question. Such codes presented a worldview of participants’ perceptual filters and lived experiences [25]. Codes were then organised into broad, open-ended themes which were identified inductively in order to remain true to the transcribed data [17,26].

After completion of the initial coding and theme development, all transcribed data were exported to NVivo Pro (v11.4.1.1064, Flexera Software LLC; Itasca, IL, USA) where codes were placed into themes and subthemes (as nodes) in order to organise and index the data in preparation for analysis. A transcribed interview sample was sent to members of the research team who were also asked to code the data and develop themes. This process was completed blindly and neither the principal investigator nor members of the research team were permitted to discuss the codes or themes that emerged from the sample during this process. The whole research team then met to discuss similarities and differences between codes, themes and data patterns and challenge the overall decision-making process of the principal investigator.

Reflexive thematic analysis was used to analyse the data with reference to the aims and objectives of the research. Themes and subthemes were refined and developed throughout analysis in a reflexive and flexible manner using a process of open coding. Themes were developed, not based on prevalence across the data set or by number of participants that articulated the data item, but rather the importance of what the data revealed about coaches’ experiences [19]. In the final stage of analysis, themes and subthemes were approved by the whole research team and cross-referenced with the aims and objectives of the study. A summary framework was developed for each theme and subtheme in order to manage and organise the large data set [27], and Stage 4 of the framework analysis guidelines proposed by Ward et al. [26] helped to organise themes into a brief summary matrix.

After reflexive thematic analysis had been agreed upon and approved by the research team, member checking was conducted to provide transparency and improve overall data trustworthiness [28]. During this process, each participant was sent their individual framework to verify that the synthesised data provided a true representation of their comments, to allow reflection on personal experiences or to add data where appropriate [28]. Last, a report was produced to detail the main findings of the study.

### 2.5. Verification

Throughout the coding process and development of themes, a detailed codebook was maintained by the principal investigator to track initial ideas, changes, or modifications to the thematic analysis process, to create an audit trail of data saturation [21], and for the purpose of reflection, traceability, and dependability [19]. Themes were adapted, updated, merged, or deleted throughout analysis and during periodic verification meetings that took place at regular and/or important stages of analysis [17,27]. Verification strategies enhance credibility, dependability, and trustworthiness during the qualitative process (terms analogous to reliability and validity in quantitative research) and help to reduce potential bias from the research team [29]. This is important in realist approaches to thematic analysis [17]. Additionally, blinded sampling of data was implemented early in the analysis process to enhance dependability and interpretation of codes and themes, and final member checking was performed to help improve transparency and trustworthiness [28].

### 2.6. Reflexivity

To strengthen the credibility and transferability of this research from theory to practice (and to deepen the overall understanding of the research question), the principal investigator utilised the practice of *reflexivity* throughout the research process [20]. As such, the following information should be used to contextualise and appraise the credibility of this study, as well as to assist in its transparency.

The primary research question originated as part of a wider investigation into strength sports as part of a Philosophy Doctorate and aimed to investigate the complex topic of OT/OTS from the perspective of the strength sport coach; an area of applied research that has not yet been explored to date, and as such, lacks qualitative analysis. Improved communication between laboratory research and applied experience is key for the development of optimal coaching practices, particularly in underinvestigated areas such as OT/OTS where the existing literature suggests dichotomy between experimental research and the application of such evidence in the training environment [10,12,13,14,15]. The principal investigator has practical experience and a research interest in strength sports, and, as such, understands the importance of advancing knowledge by bridging the gap between research and practice. During the development of the interview guide and subsequent data collection and analysis stages, the principal investigator aimed to distinguish their own experiences and perceptions from those held by participants in order to remain objective; an aspect of qualitative research essential within realist practice [30].

## 3. Results

**Figure 2:**
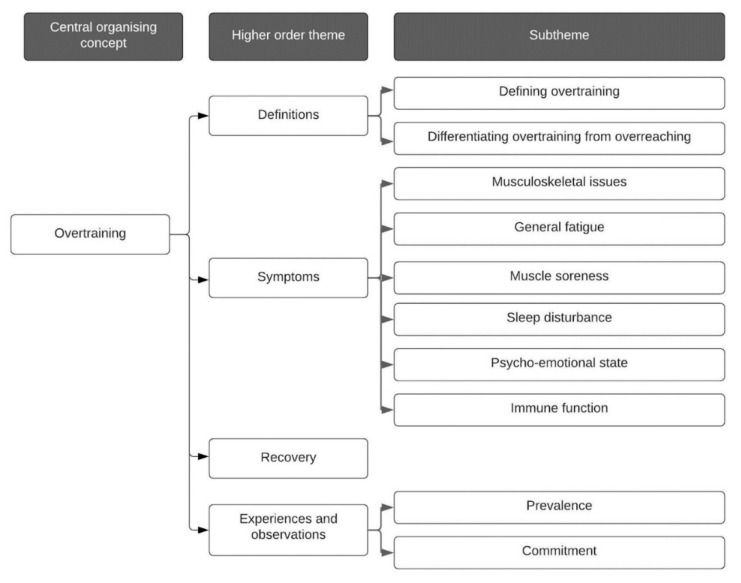
Schematic representation of central, higher order and subthemes.

Interviews were conducted between July 2019 and March 2020 and lasted between 0:39:44 and 1:17:38 min (mean 0:57:01; SD = 0:10:27 min). Audio recordings were transcribed verbatim, resulting in 276 pages of data (mean = 19.7; SD = 4.4 pages). A central concept of *overtraining* was organised into four higher order themes, with subthemes developed to further organise the large volume of data collected and to aid in the publicising of information. Figure 2 provides a schematic representation of themes and subthemes. Direct, anonymised quotes were used within the main report to illustrate discussion points and to contextualise participant experiences. Additional words were placed in parentheses to clarify intended meaning or provide further context where required. Punctuation was also added to quotations to reduce ambiguity where relevant.

### 3.1. Definitions

#### 3.1.1. Defining Overtraining

In this theme, 11 participants (1–9,11,12) characterised OT as a miscalculation of training load resulting in maladaptation and/or suboptimal response to training. OT was associated with either short- or long-term performance decline as well as loss of motivation. Synonyms were frequently used to describe the complex, multifactorial nature of OT, which included the terms *“under-recovery”* (1) and “*fatigue syndrome”* (7), however, the term *overtraining syndrome* was not used by any participants at any time.

***“So, if as part of the rebound... as part of the recuperation week they don’t rebound back to where you expect, you can term that overtraining I guess”***

(1).

***“I guess it would just be being flat-lined or turning in the opposite direction”***

(4).

***“I would say (overtraining) is a systematic decrease in the capacity of the athlete to either endure training... a decrease in the outcomes of desired adaptations and a decrease in performance over a period of time”***

(11).

***“Overtraining for me is generally not listening to your body”***

(7).

For one participant (5), the term “*overtraining*” was used to define the *act* of training above a recovery threshold. Participant 11 viewed OT as the “*capacity of the athlete to endure training*” alluding to OT as a verb to describe training, rather than a multifactorial condition as observed with OTS.

***“Feeling bad for a week or two weeks or three weeks, that’s like the colloquial ‘overtraining’… you’re doing too much”***

(5).

For some coaches (3,4,9,12) OT was an ambiguous concept and difficult to define.

***“Honestly, that’s a real difficult one”***

(9).

#### 3.1.2. Differentiating Overtraining from Overreaching

Overall, OT was seen as a progression from OR caused by prolonged exposure to the training stimulus. OT was considered an unwanted “*deviation*” (1) from planned programming resulting in unwanted symptoms of fatigue and performance decline, whereas OR was described as a deliberate, intentional part of the training process. For some (1,2,4), “*intention”* and “*outcome”* were what distinguished OR from OT.

***“I guess there’s a fine line between overreaching and overtraining, and the difference will be the ability to rebound and bounce back in a short period of time...if it comes to the point where it means you have to deviate from the plan... if you overreach, it’s part of the plan, you overtrain you’ve gone too far”***

(1).

### 3.2. Symptoms

In this theme, participants characterised OT by associated or indicative symptoms that either result in an overtrained state or indicated risk of OT. Overall, several symptoms were provided by participants as possible identifiers of OT. These ranged from physiological manifestations such as musculoskeletal issues, fatigue, and muscle soreness to psychological symptoms of altered mood state, loss of motivation and reduced readiness to train.

#### 3.2.1. Musculoskeletal Issues

One of the most commonly cited symptoms of OT related to musculoskeletal issues caused by *“overuse”* (1,3,6,7,10,11). Participants revealed that OT might result in musculoskeletal maladaptation specifically in muscle, joint and/or connective tissue. Terms such as *“tendinopathy injuries”, “physical knocks”* and *“niggles”*, were used to describe manifestation of OT, particularly within shoulders, elbows, hips, lower back, or knees.

***“But when you start seeing someone as overtrained, or trained particularly in a poor way, you start seeing issues with tendon, ligament and bone deformities”***

(11).

***“Well, the most common (symptoms) tend to be like the sore elbows, the sore knees, that kind of thing. And they’re often just a function of people doing too much, way too soon, or when their body isn’t really primed for it”***

(3).

#### 3.2.2. Psycho-Emotional State

Terms including *“apathy towards the sport”* (3)*, “abnormal behaviour”* (6), *“irritability”* (8), *“mood swings”* and *“(lack of) compliance”* (9) were cited as OT symptoms by participants 3,6,8–10. Psycho-emotional state was associated with diminished readiness to train, and participants discussed these manifestations independent of physiological disturbances or alterations in performance parameters.

***“The definition of overtraining really is to see somebody come in the gym with no motivation at all. It can be other reasons, but if their lifestyle hasn’t changed at all, and they’re coming in(to) the gym and they can’t be bothered... that’s overtraining I think”***

(8).

#### 3.2.3. General Fatigue

Four coaches (7,8,10,12) considered fatigue to be an indicator of OT, suggesting signs of “*sluggishness*”, *“lethargy”* (10), and “*extreme fatigue*” (6) could indicate chronic maladaptation. These were linked, albeit not explicitly in some cases, to reduced physical performance. For some coaches, multidimensional references to fatigue were contextualised either as reduced physical function (for example decreased strength) or mental capacity and/or emotional state.

***“I think where somebody shows signs of extreme fatigue. That’ll be displayed in a bit more abnormal behaviour. So, they might be a bit more sluggish in getting the weights on the bar and actually lifting the weights”***

(6).

***“Suddenly their bodies were like, ‘I can’t do this’. And so, there was a real peak in strength and then within a week, dropped massively”***

(7).

#### 3.2.4. Muscle Soreness

For participants 7,10,12, muscle soreness was an important indicator of OT. For coach 8, muscle soreness was expected during periods of increased training demand; but needed to be distinguished from the muscle soreness experienced during OT.

***“I’d imagine they would talk about that soreness; their readiness would go down; they’d talk about not being able to lift the weight they were doing previously”***

(10).

***“Does it (overtraining) exist in strength sport?... I think in the powerlifting sport, I think it’s more the DOMS and peripheral fatigue”***

(8).

Differentiating between muscle and joint soreness was important for coach 12, resulting in either the continuation, or cessation of exercise training.

***“If your muscle is sore, get over it. But if your joint is hurting... there’s a difference between soreness and ‘it hurts’. If it’s hurting, I will stop the session or I will change to do something else... because there’s always something else you can do”***

(12).

#### 3.2.5. Sleep Disturbance

Disturbed sleep was strongly associated with OT by participants 8 and 10, either as a direct cause of OT, or as an indirect effect. In both cases, poor sleep was related to impaired physical capacity.

***“I think (it) was maybe a bit of fatigue and that messed up his sleeping pattern. He just sort of couldn’t switch off because his body was... I can’t remember what he said. He said he felt twitchy or something with it.”***

(10).

#### 3.2.6. Immune Function

For three participants (3,8,10) symptoms of compromised immune function (or the development of acute illness) or increased prevalence of acute upper respiratory tract infections could be used to indicate the risk of OT, presenting either concurrently with psychophysiological symptoms, or independently.

***“And if a lifter’s starting to get regular colds and that, it could be that the system is being overloaded too much”***

(8).

### 3.3. Recovery

In this theme, seven participants (1,2,4,6,9,10,13) revealed the duration they considered necessary to recover from OT. For most (1,2,6,9,10,13), a period of days to two weeks would be required, but for one (4), convalescence would require months or years.

***“You know, some people can bounce back within four days, some people take two weeks, but we never see much beyond those two different extremes”***

(9).

***“Things that they say like, “oh, yeah, I just can’t be arsed today”. But it’s not just one day they say that, it’s a couple of days or a week or something and it starts to become a bit of a pattern or they say… and then you just don’t see them for a few days.”***

(6).

***“In terms of strength sports. I think overtraining is when, just by having an acute recovery period of, you know, one, two or three weeks of minimal training, it brings it back to pretty much baseline. They can go again”***

(2).

***“I’d say probably 18 months to 2 years”***

(4).

### 3.4. Experiences and Observations 

In this theme, all participants (1–14) discussed their experience of OT. Most (2–6,8–12,14) had not observed OT, and some considered terminology relating to OT and OTS to be overused, and hard to distinguish from *“a lot of moaning”* (6).

#### 3.4.1. Prevalence

The majority of participants had not observed OT. One participant (12) had *“heard of people”* (12) exhibiting symptoms of OT, and another participant (7) revealed they “*possibly*” observed an athlete OT that led to a subsequent recovery period lasting 6–9 months. Interestingly, participant 7 referred to this occurrence as OR and not OT or OTS.

***“I make it very clear that the medical overtraining I have not seen with strength sport athletes”***

(5).

***“I haven’t seen anyone experience it”***

(3).

***“From a performance perspective, no”***

(2).

***“Overtrained? I’ve heard of people... I don’t know of anybody directly myself.”***

(12).

***“I don’t think I’ve experienced overtraining… just doing too much powerlifting is very, very hard to do unless you are doing something insane with max testing all the time… because powerlifting just isn’t that much work”***

(6).

***“It’s not something I see often, or barely at all”***

(14).

OTS might exist according to participant 8, but is unlikely within the strength sport domain, elaborating that some athletes could work at a greater relative load/intensity and still achieve performance improvements without the risk of OT/OTS developing.

***“So, I think it does exist, but I don’t see many lifters, my lifters, who are really overtraining to be honest… and I think some could push themselves a bit harder”***

(8).

#### 3.4.2. Commitment 

Four participants (5,6,10,12) discussed the relationship between training hard and being a successful athlete. For coach 12, a blurred line between commitment and OT exists. For coach 5 though, a *“train at all costs”* attitude could be considered a risk factor for OT.

***“We had this mentality instilled in these kids from an earlier age of you have to work hard. The hardest worker or the best. There’s this, this valuation of effort over results”***

(5).

***“Is it overtraining or just work ethic?”***

(12).

## 4. Discussion

The aim of this study was to examine high-performance strength coaches’ experiences of OT/OTS and to provide a new way of understanding and conceptualising these concepts in strength sports. Results of this investigation provide important contextual evidence of training maladaptation from the perspective of the strength sport coach; an area of research not yet explored in this domain. Findings demonstrated that strength sport coaches typically revealed different experiences and understanding of OT/OTS and are unaware of expert consensus literature. Findings also note the importance of collaboration between researchers, academics and coaches when developing a holistic approach to understanding OT/OTS.

### 4.1. How Did Coaches Define Overtraining?

Definitions between OT and OTS were interchangeable. Participants described aspects of training maladaptation in a detailed but diverse way, with little to no reference to accepted terminology provided by expert consensus. Similarly, there was a lack of cognisance relating to accepted diagnostic criteria used for the objective identification or diagnosis of OTS. Moreover, participants seemed unaware that these existed. On occasion, participants did, albeit indirectly, acknowledge the difference between OT and OTS, using terms such as “physiological OT” and “fatigue syndrome” to describe training maladaptation indicative of OTS. However, the term “overtraining syndrome” was not used by any participant at any time. “Overtraining” on the other hand was used colloquially to refer to the *act* of training excessively. OT was differentiated from OR based on outcome, with OR considered a planned and intentional training tool where short-term periods of elevated training demand resulted in a positive training outcome, and OT being a training error resulting in maladaptive response. The terms FOR or NFOR were not used at all by any participant.

Short-term increases in training demand can induce functional overreaching (FOR) leading to improved physical capabilities. However, NFOR or the OTS occur when high training-demand is undertaken for extensive periods without sufficient recovery. In this case, it is the time-course to recovery from impaired performance that provides the distinction between NFOR and OTS; NFOR requires *weeks to months* of performance restoration, whilst OTS can require *several months* [10,16,31]. Previous research has accepted that a lack of consistent terminology and definitions is a major concern for understanding the aetiology of OTS, and that a lack of consistency might hinder both the ability to compare results of research studies and apply such findings in practice [11,15,16]. This is illustrated in the literature by the use of the term *overtraining* when referring to both the prescription of a short-term resistance training protocol where no incidence of OTS was reported [32], and also to describe training protocols purposely designed to induce OTS [33]. Divergent use of terminology elucidates confusion when determining OT and OTS and can lead to potential misdiagnosis [11]. In an attempt to improve the understanding of OTS, broader, alternative terminology has been proposed, such as the “unexplained underperformance syndrome” [11] and “paradoxical deconditioning syndrome” [34]. These have failed to gain traction within the field but do demonstrate the multiple attempts made to address terminology of OT/OTS. Such varied terminology may be a contributing factor for the range of definitions provided by high-performance coaches in this study.

### 4.2. How Prevalent Did Coaches Consider Overtraining to Be?

Participants of this study considered prevalence of OT/OTS to be low; with few observing or encountering overtrained athletes in their respective sport. Moreover, many participants considered it unlikely that strength sport athletes would experience chronic maladaptation indicative of OTS. In most cases, participants seemed unconcerned about OT/OTS and at times suggested athletes could work at a higher relative load/intensity than is typical of their current training, without deleterious effects.

Studies from endurance sports have elucidated NFOR/OTS prevalence to be 7–21% during a training monocycle, although it is acknowledged that these figures are estimates [16]. The evidence for prevalence of OTS within both strength sports and resistance training is very low, with only limited cases reported [12,14]. However, a lack of experimental research may be a contributing factor to such low prevalence, and further exploration will help to inform more clearly the dichotomous findings between strength and endurance populations. Cross-sectional survey research has demonstrated that symptoms of unexplained underperformance reported by competitive strength athletes typically last for only short periods of time (1 week to 1 mo = 43.8%), with fewer reporting training maladaptation indicative of NFOR (1–3 mo = 13.1%) or OTS (>4 mo = 4.7%) [15]. However, whilst this study [15] provides important contextual information relating to training maladaptation and prognostic symptoms of OTS from the perspective of the strength sport, caution should be advised when interpreting these results. Response and recency bias may impact self-reporting of symptoms. Moreover, this study did not control for responder misinterpretations of “unexplained underperformance”, and as such it is possible that conditions relating to low energy availability or incidence of acute illness may have contributed towards such symptoms. Further experimental research is needed to accurately elucidate the prevalence, symptoms, and experiences of NFOR/OTS within strength and resistance sport contexts.

### 4.3. What Symptoms Did Coaches Associate with Overtraining?

Several symptoms were perceived as indicators of OT/OTS by participants of this study. For a small number of participants, performance change was an important manifestation of OT/OTS, whereas for others, performance outcome was less important. Overall, references to performance were often not explicit; instead, performance alterations were contextualised within discussions relating to other symptoms: physiological manifestations such as overuse issues and increased muscle soreness, or psychological symptoms such as reduced readiness to train or altered psycho-emotional state. The most frequently cited symptoms provided by participants of this study related to musculoskeletal injury as a result of overuse, and symptoms of general fatigue.

NFOR and OTS present with or without physiological and psychological symptoms [10,16] and several proposed biochemical and pathophysiological markers have failed to prospectively elucidate OTS [16,31]. Moreover, common symptoms exhibited in overtrained endurance athletes might not be indicative of those exhibited within strength sport athletes [10,13]. Previous research has highlighted risk factors, symptoms and mechanisms that may assist in the detection of NFOR/OTS within strength sports and resistance training, however, no single diagnostic tool has been determined, and further research is required [12,14,15]. It is accepted that aerobic and resistance exercise protocols can elicit different biological responses, likely due to the contrasting adaptations created by each mode of exercise. This may explain, in part, why both the symptomatic profile and response to NFOR/OTS might differ between strength and endurance athletes [12,13,14].

Risk factors for the onset of NFOR/OTS in strength sports include the continued pursuit of high-intensity or high-volume and monotonous resistance training and/or training to muscular failure, as well as prolonged low energy availability and/or carbohydrate consumption [12,14,15]. Several performance, neuromuscular, and biochemical mechanisms have been proposed as markers to determine NFOR/OTS in both strength sports and resistance exercise, but no single test or method has yet been able to identify either state [12,14]. Further investigation using well-designed protocols, under both experimental conditions and within an applied context, will help to reduce the negative impact of NFOR/OTS on strength performance.

Impaired muscle recovery and dysfunctional muscle response have been suggested as predictive characteristics of OTS [35]. However, musculoskeletal issues resulting from high training demand might be the result of acute exercise-induced tissue microtrauma leading to acute training maladaptation or the cumulative effects of OR rather than OTS [15,36], therefore, caution is advised when diagnosing OTS based on the presence of musculoskeletal issues alone. However, coaches should monitor training load for manifestations of musculoskeletal aches and pains regardless, to ensure optimal adaptation to resistance exercise and to reduce risk of orthopaedic injury, as this can be equally as debilitating as OTS and both would require substantial recovery time [10].

Whilst performance decline is considered the “gold standard” symptom of OTS, decreased performance and/or increased perceived effort at a given relative intensity are also typically observed during periods of acute fatigue and FOR [15]. Premature reductions in training based on the presence of short-term performance decrement or in the presence symptoms of general fatigue might lead to a miscalculation in training. Currently, OTS can only be diagnosed in the presence of prolonged maladaptation leading to a decrease in performance during training or competition lasting several weeks to months [10,16], and whilst a decline in performance associated with OTS is likely to be accompanied by symptoms of generalised fatigue, the presence of fatigue in itself is not necessarily synonymous with OTS [15]. Therefore, measures of generalised fatigue should only be included within a pragmatic, multidimensional diagnostic tool when used to identify OTS.

Participants in this study made occasional reference to a possible causal relationship between OT and disturbed sleep. Sleep loss can have a significant detrimental effect on readiness to train, motivation, cognition, and sports performance, and as such, monitoring of sleep has been proposed as a useful tool for early detection of performance decrement [37]. Additionally, stress and anxiety caused by increased training demand and/or competitive schedule, as well as stress caused by underperformance, can result in sleep disturbance [38]. Furthermore, sleep is an essential component of restoration and recuperation, therefore, it is feasible that sleep loss could also accelerate symptoms of NFOR/OTS by reducing recovery capacity during periods of increased training demand [38]. However, it has also been argued that a relationship between NFOR/OTS and sleep disturbance could be coincidental [39]. Further research in this area will elucidate the possible relationship between sleep disturbance and OTS, or the validity of sleep loss as a predictor of OTS.

### 4.4. How Long Did Coaches Consider Was Necessary to Recover from Overtraining?

Perhaps the most dichotomous thinking between participants and accepted expert consensus related to time needed for performance restoration following symptoms of OT. For many participants, recovery from OT necessitated days to ≤2 weeks of recovery, with only a single reference to longer convalescence periods. The general consensus from participants was that whilst OT was associated with under-recovery and reduced motivation to train, such symptoms lasted only for a short duration of time. 

Current expert consensus suggests that OTS is characterised by the persistence of underperformance lasting several weeks to months, or in some cases even longer [10]. Moreover, that NFOR requires several days to weeks to recover from. By this rationale, participants elucidated a restorative time-course more analogous to acute fatigue or OR, but not OTS. Of course, it has already been discussed that participants in this study identified OT more as the *act* of participating in high-demand training, and displayed little understanding of true OTS, which could in part, help to explain the reference to shorter recovery durations. This further illustrates the lack of awareness and understanding of accepted terminology.

## 5. Practical Applications

This study provides important contextual evidence relating to the understanding of training maladaptation from the perspective of the high-performance strength sport coach; an area of research not yet explored in depth. The inconsistent interpretations provided by participants demonstrates the need for the provision of accessible information in a way that allows accurate identification of NFOR and/or OT/OTS within a practical setting, thus facilitating the successful planning and organisation of training and performance.

Previous literature has highlighted the importance of an *evidence-informed* approach to high-performance sport science where collaboration between researchers and stakeholders (including coaches and support staff), is key for the development of best practice [40]. This study has indicated (within the high-performance strength sport domain), that coaches are at times unfamiliar with the underpinning terminology, concepts and paradigms of OT/OTS. Thus, a more robust, iterative system of knowledge translation may be required to help strength sport coaches understand the nuances of OT/OTS in order to support decision-making, and thus bridge the gap between research and applied practice. Whilst academics rank peer-reviewed journals highly when obtaining scientific evidence, coaches and high-performance support staff are more likely to report a greater propensity towards informal communication [41]. Consequently, the development of coach educational resources, peer discussion and discussion relating to shared experience may be a viable option to improving the understanding of research to practice for OT/OTS.

## 6. Study Strengths and Limitation

Coaches provided real life insight and experiences of OT/OTS that will underpin and develop further understanding within applied practice. This study is the first to analyse information about experiences of OT/OTS within a group of strength sport coaches and highlights current understanding and worldview within this group. Moreover, this study utilises a qualitative analytical method that, whilst flexible and reflexive, allows for analysis in a systematic and precise way. The findings of this study should serve as a catalyst for further investigation in this area of research. 

Whilst this study offers new insight into OT/OTS, it is recognised that limitations do exist. Inclusion criteria ensured that the sample of participants derived from high-performance strength sports, thus creating a level of homogeneity. Future research may benefit from analysing coaches’ perspectives from amateur athletic populations as well as from a broader scope of sports (intermittent, concurrent or endurance) which has not yet been performed. Recruitment followed an opportunity, snowball approach, with a heavy reliance on social media distribution. Whilst this provided a fair and unobtrusive recruitment strategy, it might also have biased participants who met all necessary criteria and were quick to respond to recruitment information. It might also have excluded those who were eligible to participate, suitably experienced and appropriately qualified, but not attached to social media communication, and therefore unaware of the opportunity.

## Figures and Tables

**Table 1:** Descriptive characteristics of participants.

Participant	Sex	Strength Sport	Location	Experience (Years)	Experience Level
1	Male	Weightlifting	UK	20	International
2	Male	Powerlifting	IRE	6	International
3	Male	Powerlifting	IRE	5	International
4	Male	Weightlifting	UK	9	International
5	Male	Powerlifting	USA	10	National
6	Male	Weightlifting	UK	12	International
7	Female	Weightlifting	UK	4	International
8	Male	Weightlifting	UK	57	International
9	Male	Powerlifting	UK	15	International
10	Male	Throws	UK	15	International
11	Male	Sprints	UK	10	International
12	Female	Sprints	UK	4	International
13	Male	Jumps	UK	13	International
14	Male	Throws	NZ	21	International

UK = United Kingdom; IRE = Ireland; USA = United States of America; NZ = New Zealand.

## Data Availability

Data sharing not applicable.
